# Endovascular thrombectomy for acute ischemic stroke: evolving patient selection, procedural strategies, and adjunctive therapies

**DOI:** 10.3389/fneur.2026.1861577

**Published:** 2026-08-04

**Authors:** Jie Xu, Jing Bao, Shepeng Wei

**Affiliations:** Department of Neurosurgery, Shanghai Shidong Hospital of Yangpu District, Shanghai, China

**Keywords:** acute ischemic stroke, endovascular thrombectomy, intra-arterial thrombolysis, medium-vessel occlusion, patient selection

## Abstract

Endovascular thrombectomy (EVT) has transformed the treatment of acute ischemic stroke caused by large-vessel occlusion; however, its expanding indications require increasingly precise patient selection and procedural execution. This mini-review summarizes contemporary issues in EVT that go beyond the original early-window anterior-circulation paradigm. Current evidence supports the use of EVT for selected anterior-circulation large-vessel occlusion, late-window stroke, large-core infarction, and basilar artery occlusion, while more marginal scenarios—including a very low Alberta Stroke Program Early CT Score (ASPECTS), minor stroke with large-vessel occlusion, presentations beyond 24 h, prestroke disability, and medium-vessel occlusion—require individualized assessment. Recent randomized trials have tempered enthusiasm for routine thrombectomy in medium-vessel occlusion, emphasizing the need to distinguish technical accessibility from meaningful clinical benefit, particularly when eloquent brain regions are involved. Procedural strategy has also shifted from simple recanalization toward high-quality, tissue-effective reperfusion, with attention to the access route, device selection, first-pass effect, reperfusion grade, tandem lesions, intracranial atherosclerotic disease, anesthesia, antithrombotic use, and blood-pressure management. Adjunctive therapies, especially intra-arterial thrombolysis after successful, incomplete, or failed reperfusion, remain promising but should be employed selectively rather than as a routine practice. Overall, the future of EVT lies in disciplined precision: selecting patients for whom reperfusion is likely to be beneficial, optimizing procedural quality, and developing adjunctive strategies that convert angiographic success into durable functional recovery.

## Introduction

Acute ischemic stroke remains a time-sensitive emergency in which the outcome for affected tissue depends on the rapid restoration of blood flow (1, 2). For eligible patients within 4.5 h of last known well state, intravenous thrombolysis with alteplase or tenecteplase in appropriate settings remains a standard therapy and should not delay the preparations for endovascular treatment (1). Over the past decade, endovascular thrombectomy (EVT) has become a standard treatment for anterior-circulation large-vessel occlusion, with evidence now extending to broader time windows, larger infarct cores, selected posterior-circulation occlusions, and more complex clinical scenarios (1, 2). However, EVT remains applicable to only a minority of patients with ischemic stroke, and access is still limited due to the concentration of thrombectomy-capable services in specialized centers (3, 4). Therefore, rapid triage, interhospital transfer, and the integration of intravenous thrombolysis with EVT pathways remain essential (5).

**Figure 1:**
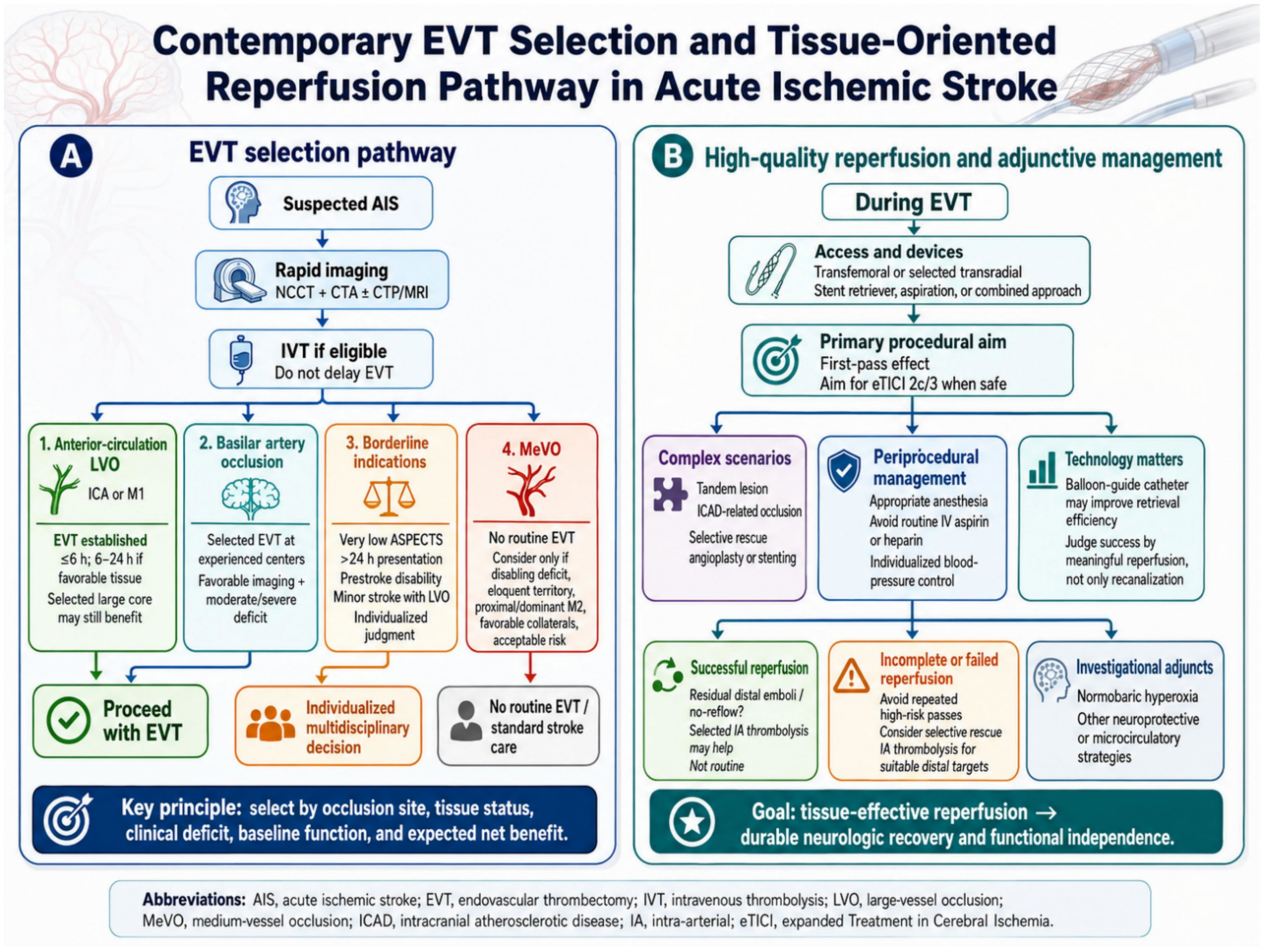
Contemporary EVT selection and the tissue-oriented reperfusion pathway in acute ischemic stroke. **(A)** EVT selection pathway. **(B)** High-quality reperfusion and adjunctive management during EVT. The pathway emphasizes integrated patient selection based on time window, occlusion site, infarct burden, collateral status, clinical deficit, prestroke function, and procedural risk. EVT is established for selected anterior-circulation large-vessel occlusion and is increasingly supported in selected large-core infarction and basilar artery occlusion. In contrast, very low ASPECTS, presentations beyond 24 h, minor stroke with large-vessel occlusion, prestroke disability, and medium-vessel occlusion require individualized judgment. Procedural strategy should aim for rapid, durable, and near-complete reperfusion, with selective use of rescue angioplasty or stenting in tandem lesions or intracranial atherosclerotic disease-related occlusion. Adjunctive therapies, including intra-arterial thrombolysis and normobaric hyperoxia, remain selective or investigational approaches to improving tissue-level reperfusion.

As the indications for EVT expand, the key question is no longer simply whether EVT works, but how to identify patients in whom reperfusion is likely to produce meaningful recovery. This mini-review focuses on three current issues: evolving patient selection, including late-window stroke, large-core infarction, posterior-circulation occlusion, minor stroke with large-vessel occlusion, and medium-vessel occlusion; procedural strategies aimed at improving first-pass and near-complete reperfusion; and adjunctive therapies, particularly intra-arterial thrombolysis after successful, incomplete, or unsuccessful reperfusion. We frame EVT not merely as arterial opening, but as a tissue- and outcome-oriented reperfusion strategy. A schematic overview of this contemporary EVT selection and tissue-oriented reperfusion pathway is provided in Figure 1.

## Expanding patient selection: from time windows to tissue and clinical context

Patient selection is now central to contemporary thrombectomy. As EVT indications have expanded, decision-making has moved from rigid time- and score-based eligibility toward an integrated assessment of occlusion site, infarct burden, collateral status, clinical severity, prestroke function, and the likelihood that reperfusion will translate into meaningful recovery (1, 2). The goal is not merely to identify technically recanalizable arteries, but to distinguish between patients for whom reperfusion is likely to preserve functionality and those for whom it may be futile or even harmful.

For anterior-circulation large-vessel occlusion, including selected late-window and large-core presentations, EVT is firmly established, particularly for intracranial internal carotid artery or M1 occlusion (1, 2). Intravenous thrombolysis should be given when indicated, but EVT should not be delayed to observe the response to thrombolysis (1). The strongest early evidence stems from pivotal trials and the HERMES individual-patient meta-analysis, which examined patients treated within 6 h (6). DAWN, DEFUSE 3, and AURORA subsequently shifted late-window selection from a purely time-based paradigm toward tissue-based eligibility (7–9).

Large-core infarction has further changed the interpretation of infarct burden. Recent randomized trials and subsequent evidence syntheses indicate that patients with larger established infarcts can still benefit from EVT, although the absolute likelihood of functional independence is lower and many patients remain disabled despite treatment (10, 11). ASPECTS remains a pragmatic surrogate of infarct extent, particularly when advanced imaging is unavailable; however, it should be interpreted as one component of decision-making rather than as a rigid threshold (12–14). At the lower end of the ASPECTS range, especially ASPECTS 0–2, evidence remains limited, and the risks of hemorrhagic transformation and futile reperfusion must be weighed carefully; nevertheless, current imaging tools may incompletely distinguish irreversibly injured tissue from tissue that may still be salvageable (15, 16).

Advanced perfusion imaging with CTP or MRI remains useful for estimating the ischemic core and salvageable tissue in late-window presentations (17, 18). However, automated perfusion-core estimation is not mandatory for all patients, particularly when software is unavailable or would delay treatment (19). Non-contrast CT, CTA-defined occlusion site, and collateral assessment offer a pragmatic alternative, supported by MR CLEAN-LATE and its 2-year follow-up (20, 21).

Posterior circulation and individualized decision-making: Posterior-circulation stroke illustrates why EVT selection cannot simply copy anterior-circulation criteria. EVT is appropriate for selected patients with basilar artery occlusion, especially when their neurological deficits are moderate to severe, baseline imaging remains favorable, and treatment can be delivered within 24 h at experienced centers (1, 22). This position is supported by randomized trials on basilar artery occlusion, including ATTENTION and BAOCHE (23, 24). The VERITAS individual-patient-data meta-analysis also supports improved functional outcomes and reduced mortality with EVT in vertebrobasilar occlusion, but the evidence remains more context-dependent than in anterior-circulation LVO (22). Stroke mechanism, collateral anatomy, infarct tolerance, intracranial atherosclerotic disease, and use of adjunctive angioplasty or stenting may all influence treatment effects. By contrast, routine EVT for isolated vertebral artery or posterior cerebral artery occlusion remains insufficiently supported, although selected disabling presentations may justify case-by-case consideration (22, 25).

Marginal indications for EVT include very low ASPECTS, minor stroke with LVO, and MeVO. The most difficult decisions now occur at the margins of benefit. EVT is less likely to be useful when there is extensive established infarction, minimal salvageable tissue, severe prestroke disability, limited life expectancy, or when the clinical context makes technical reperfusion unlikely to produce meaningful recovery (2, 26). However, these factors should be understood as modifiers of expected benefit rather than automatic exclusion criteria. Contraindications to intravenous thrombolysis, including selected cases of infective endocarditis, do not necessarily preclude EVT when the occlusion, imaging profile, and clinical context are otherwise favorable (27). Similarly, patients presenting beyond 24 h may occasionally be considered when imaging suggests persistent salvageable tissue, although this remains outside the strong randomized evidence base (28, 29).

Minor stroke with LVO is another unresolved boundary. Low NIHSS scores should not be equated automatically with low clinical impact, because aphasia, hemianopia, neglect, or disabling hand weakness may substantially affect independence despite a numerically mild score (30, 31). Treatment decisions should therefore depend on whether the deficit is functionally disabling, whether the occlusion is likely to deteriorate without reperfusion, and whether expected benefit outweighs procedural risk.

MeVO represents a particularly important boundary between technical feasibility and proven clinical benefit. ESCAPE-MeVO and DISTAL did not show an overall functional benefit of routine EVT for MeVO or medium/distal vessel occlusion (32, 33). These findings argue against routine thrombectomy for MeVO as a class, while still leaving room for carefully selected patients with disabling deficits, proximal or dominant M2 occlusion, eloquent-territory involvement, favorable collaterals, limited infarct burden, and acceptable procedural risk (32–34). Because MeVO raises distinct questions regarding trial interpretation, eloquent brain regions, procedural risk, and patient-centered outcomes, it is discussed separately below.

## Medium-vessel occlusion: technical feasibility versus clinical benefit

MeVO has become a critical test of whether the thrombectomy paradigm can be extended beyond classic proximal LVO without mistaking technical reachability for clinical indication. These occlusions are increasingly recognized on CTA and may involve the M2 or M3 branches of the middle cerebral artery, the A2 or A3 segments of the anterior cerebral artery, P2 or P3 segments of the posterior cerebral artery, or other medium-sized intracranial vessels. Although MeVOs usually involve smaller clot burden and more distal territories than ICA or M1 occlusions, they may still cause disabling cortical syndromes when dominant or eloquent regions are affected. The key question is therefore not whether the vessel can be reached, but whether intervention improves patient-centered outcomes enough to justify procedural risk.

Recent randomized trials have tempered enthusiasm for routine EVT in MeVO. In ESCAPE-MeVO, EVT plus usual care did not improve 90-day functional outcomes compared with usual care alone in patients treated within 12 h (32). In DISTAL, EVT plus best medical treatment likewise failed to reduce disability or death in patients with isolated medium or distal vessel occlusion treated within 24 h (33). These neutral results challenge the assumption that a visible, technically removable clot necessarily represents a clinical indication for thrombectomy. They also show that procedural feasibility, angiographic success, and biological plausibility are not substitutes for demonstrated functional benefit.

Several factors may explain why MeVO trials have not reproduced the treatment effect seen in classic LVO trials. MeVO patients often have lower NIHSS scores, smaller infarct volumes, more heterogeneous clinical syndromes, and a more favorable natural history than patients with proximal LVO (32–34). The absolute room for benefit may therefore be smaller. At the same time, distal thrombectomy is technically more demanding: smaller, more tortuous, and more fragile vessels increase the risk of perforation, dissection, vasospasm, distal embolization, and hemorrhagic transformation, especially after repeated device passes. Even modest procedural risk may offset modest expected clinical gain.

However, neutral trial results should not be interpreted as proof that no patient with MeVO can benefit from EVT. MeVO is not a homogeneous category. A proximal dominant M2 occlusion causing severe aphasia is clinically different from a distal M3 occlusion causing mild sensory symptoms. Similarly, occlusions involving the language network, motor hand area, visual cortex, or other eloquent regions may be poorly captured by a low NIHSS score. Thus, MeVO decision-making should be function-oriented rather than vessel-diameter-oriented.

Current evidence argues against routine EVT for MeVO as a class, but leaves room for carefully selected intervention in patients with clearly disabling deficits, proximal or dominant M2 occlusion, small established infarct, favorable collaterals, limited procedural complexity, and acceptable hemorrhagic risk. Conversely, routine intervention is difficult to justify for mild non-disabling symptoms, very distal occlusions, poor collaterals, extensive infarction, or anatomy likely to require repeated high-risk manipulation. Future studies should refine selection by integrating occlusion dominance, eloquent-territory involvement, infarct volume, collateral status, device strategy, first-pass reperfusion, and patient-centered outcomes. Until then, MeVO should remain an area of disciplined restraint rather than automatic expansion.

## Procedural strategy: from recanalization to high-quality reperfusion

If patient selection determines who should undergo EVT, procedural strategy determines whether reperfusion is achieved safely, efficiently, and in a form that translates into neurologic recovery (2). The modern procedural goal is not simply arterial opening, but fast, complete, durable, and tissue-effective reperfusion while minimizing embolization, vascular injury, hemorrhagic transformation, and treatment delay (35–37).

Devices, access, first-pass effect, and reperfusion grading. EVT is most commonly performed through transfemoral access, although transradial access is increasingly used in selected patients with unfavorable arch anatomy, iliofemoral disease, obesity, anticoagulation, or higher access-site bleeding risk (38). The access route should be guided by speed, catheter stability, vascular anatomy, device compatibility, and operator experience, rather than by a universal preference for one approach. Similarly, device strategy should remain flexible. Stent retrievers, aspiration catheters, and combined approaches each have theoretical and practical advantages, but randomized data do not support a single universally superior first-line technique (36, 39–42). In practice, thrombus composition, occlusion site, vessel tortuosity, cervical access, device deliverability, and the need to minimize passes should guide initial strategy and early technique switching.

The growing emphasis on the first-pass effect reflects the shift from recanalization to reperfusion quality. Near-complete or complete reperfusion on the first pass may reduce endothelial injury, distal embolization, procedure time, contrast exposure, and hemorrhagic risk. Although eTICI ≥2b remains the conventional definition of successful reperfusion, eTICI 2c/3 is increasingly viewed as the more desirable endpoint because small residual perfusion defects may still carry functional consequences (35). Balloon-guide catheters may improve clot retrieval by providing proximal flow arrest, reducing antegrade embolization, and improving retrieval efficiency (43). In an updated systematic review and meta-analysis, balloon-guided catheter use was associated with higher rates of successful reperfusion, first-pass effect, and favorable 90-day functional outcome, although the evidence remained largely observational and randomized data were lacking (43). Thus, technological progress should be judged not only by angiographic success, but by whether it improves clinically meaningful reperfusion.

Tandem lesions, ICAD, and rescue angioplasty or stenting. Tandem occlusion and intracranial atherosclerotic disease (ICAD) are complex scenarios in which thrombectomy becomes more than clot retrieval. In tandem cervical internal carotid artery and intracranial LVO, the operator must decide the treatment sequence, whether balloon angioplasty alone is sufficient, whether acute carotid stenting is required, and how to balance antiplatelet therapy against hemorrhagic risk (44–46). Acute carotid stenting may improve outcomes in selected patients, but infarct burden, thrombolysis exposure, antiplatelet strategy, and bleeding risk remain central (44–46). ICAD-related occlusion presents a different problem: residual stenosis and local thrombogenicity may lead to early reocclusion after clot removal. Rescue angioplasty, intracranial stenting, and glycoprotein IIb/IIIa inhibitors are used in practice, especially where ICAD is prevalent, but evidence remains mixed (47–49). ANGEL-REBOOT did not show improved 90-day functional outcome with bailout intracranial angioplasty or stenting and reported more arterial dissection and numerically more symptomatic hemorrhage (49). These findings argue against routine escalation, while leaving room for selected rescue therapy when durable patency is unlikely without additional treatment.

Periprocedural anesthesia and blood pressure management. Periprocedural care can determine whether technical reperfusion becomes useful reperfusion. Conscious sedation and general anesthesia can both be appropriate; the practical goal is to avoid treatment delay, hypoxia, hypercapnia, hypotension, aspiration, agitation, and patient movement (1). Antithrombotic management should also remain conservative unless there is a specific procedural indication. Routine intravenous aspirin or unfractionated heparin during EVT is not supported, because MR CLEAN-MED showed increased hemorrhagic risk without clinical benefit (50).

Blood pressure management is equally important. Before reperfusion, hypotension should be avoided to preserve collateral-dependent tissue (1, 51). After reperfusion, excessive blood pressure may promote edema, hemorrhagic transformation, and reperfusion injury, whereas overly aggressive lowering may worsen hypoperfusion in incompletely reperfused or autoregulation-impaired tissue (52). Recent randomized evidence does not support very intensive systolic blood-pressure lowering after EVT, particularly targets below 120–140 mmHg (53, 54). A more individualized approach should consider reperfusion grade, infarct size, hemorrhagic risk, baseline hypertension, collateral status, and whether recanalization is complete or incomplete. Autoregulation-guided strategies remain investigational (55, 56).

Overall, procedural strategy in EVT has shifted from device-centered recanalization to system-based reperfusion quality. The key question is which combination of access, device choice, flow control, rescue strategy, anesthesia, antithrombotic use, and hemodynamic management is most likely to deliver fast, complete, durable, and clinically meaningful reperfusion for an individual patient.

## Adjunctive therapies after EVT

Successful macrovascular recanalization does not always translate into complete tissue-level reperfusion or meaningful neurologic recovery. Even after apparently successful EVT, residual distal emboli, microvascular obstruction, impaired collateral washout, reperfusion injury, or no-reflow phenomena may continue to limit tissue salvage (57, 58). Adjunctive therapies after EVT therefore aim to close the gap between angiographic success and clinical recovery. Among these approaches, intra-arterial thrombolysis has received the greatest attention, whereas normobaric hyperoxia and other reperfusion-enhancing or cytoprotective strategies remain investigational.

Intra-arterial thrombolysis after successful reperfusion. The rationale for intra-arterial thrombolysis after successful EVT is that eTICI 2b–3 reperfusion may still leave residual thrombi in distal arterioles or the microcirculation, particularly in territories not accessible to mechanical devices (57, 58). Local thrombolytic delivery could theoretically improve downstream perfusion while limiting systemic exposure. In the CHOICE trial, adjunctive intra-arterial alteplase after successful thrombectomy improved excellent functional outcome without a clear increase in symptomatic intracranial hemorrhage (59). More recently, the PEARL randomized clinical trial also reported a higher likelihood of excellent 90-day outcome with intra-arterial alteplase after successful endovascular reperfusion (60).

However, the evidence is not uniform across thrombolytic agents. In POST-UK, intra-arterial urokinase after near-complete to complete reperfusion did not significantly increase survival without disability at 90 days (61). Similarly, POST-TNK did not significantly improve freedom from disability with intra-arterial tenecteplase after near-complete to complete reperfusion (62). Together with recent systematic and editorial assessments, these trials suggest that intra-arterial thrombolysis after successful EVT should not yet be considered routine care, but rather an evolving, agent-specific, dose-sensitive, and patient-selected strategy (63, 64).

Intra-arterial thrombolysis after incomplete or failed reperfusion. The potential role of intra-arterial thrombolysis may be different when reperfusion is incomplete or unsuccessful. In patients with residual distal emboli, inaccessible branch occlusions, or eTICI 2a–2b reperfusion, further mechanical manipulation may increase the risk of perforation, dissection, vasospasm, or distal embolization. In this setting, low-dose intra-arterial thrombolysis may be attractive as a chemical rescue strategy when the remaining thrombus is too distal or fragmented for safe device retrieval.

This indication, however, is less well supported by randomized evidence than post-successful-reperfusion use. Patients with incomplete reperfusion may have a larger infarct burden, repeated device passes, longer procedure time, endothelial injury, prior intravenous thrombolysis exposure, or blood–brain barrier disruption, all of which may increase hemorrhagic risk. Therefore, intra-arterial thrombolysis after incomplete or failed reperfusion should be framed as selective, anatomy-dependent, and risk-sensitive rather than routine. Future trials should distinguish adjunctive thrombolysis after eTICI 2c/3 reperfusion from rescue thrombolysis after incomplete or failed reperfusion, because these are biologically and clinically different scenarios.

Normobaric hyperoxia and other investigational approaches. Normobaric hyperoxia has been proposed as a simple strategy to support ischemic tissue during and after reperfusion by increasing dissolved oxygen delivery. Early randomized evidence suggested the feasibility of combining normobaric hyperoxia with endovascular treatment (65). However, the larger OPENS-2 trial did not establish normobaric hyperoxia as a routine adjunct that reliably improved clinical outcomes after EVT (66). Other investigational approaches, including neuroprotective, microcirculatory, anti-inflammatory, and imaging-guided strategies, reflect the same principle: opening the proximal artery is necessary but not always sufficient. Until patient selection becomes more precise, adjunctive therapies should be viewed as promising but unproven tools for converting angiographic reperfusion into durable functional recovery.

## Conclusion

Endovascular thrombectomy has transformed the treatment of acute ischemic stroke caused by large-vessel occlusion, but the field has moved beyond the simple question of whether thrombectomy works. The central challenge is how to extend benefits without diluting the certainty of meaningful recovery. Broader indications now include extended time windows, selected large-core infarctions, and selected posterior-circulation occlusions, but very low ASPECTS, minor stroke with LVO, prestroke disability, and MeVO still require individualized judgment.

The future of EVT will depend on disciplined precision rather than indiscriminate expansion: selecting patients in whom reperfusion is likely to matter, performing procedures that maximize fast and durable tissue-level reperfusion, and developing adjunctive strategies that convert technical success into functional independence.
